# Bruceine D attenuates prostate cancer growth and motility by suppressing PI3K/AKT signaling and downregulating CXCL16: implications for skeletal metastasis in orthopedic oncology

**DOI:** 10.3389/fphar.2026.1819854

**Published:** 2026-06-01

**Authors:** Zhiwei Chen, Qingbin Huang, Yingming Zheng, A. Garu, Xitao LingHu, Wenchong Yu, Guangquan Zhao, Jieying Zhang, Lin Tian, Yubo Tang, Shuai Huang, Xiaodong Zhao

**Affiliations:** 1 Foshan Clinical Medical School of Guangzhou University of Chinese Medicine, Foshan, China; 2 Department of Pharmacy, The First Affiliated Hospital, Sun Yat-sen University, Guangzhou, China; 3 Department of Orthopedics, The Second Affiliated Hospital of Zunyi Medical University, Zunyi, China; 4 School of Pharmaceutical Sciences, Sun Yat-sen University, Guangzhou, China; 5 The Second Affiliated Hospital of Guangzhou Medical University, Guangzhou, China; 6 The First People’s Hospital of Foshan, Foshan Hospital Affiliated to Southern University of Science and Technology, Foshan, Guangdong, China

**Keywords:** Bruceine D, cell motility, CXCL16, orthopedic oncology, PI3K/AKT, prostate cancer, skeletal metastasis

## Abstract

**Introduction:**

Prostate cancer often reaches bone, which poses a challenge in orthopedic oncology since few therapies directly suppress tumor growth in bone. Bruceine D (BD) is a quassinoid from Brucea javanica. It is known anticancer activity but its effects on prostate cancer have not been defined.

**Methods:**

In this paper, we studied BD’s drug effects on PC-3 and C4-2B prostate cancer cells using CCK-8, colony formation, wound healing, and Transwell assays. We measured apoptosis by Annexin V-FITC/PI staining and flow cytometry. RNA sequencing, Western Blotting and molecular docking were used to identify signaling pathways and potential targets. We evaluated BD’s antitumor effects and toxicities in a subcutaneous xenograft model in nude mice.

**Results:**

BD inhibited proliferation, migration, invasion and induced apoptosis in prostate cancer cells in a time- and dose-dependent manner. Transcriptomic analysis showed BD downregulated PI3K/AKT pathway components (PIK3AP1, PIK3C2B, PIK3CB, PIK3R2, AKT1) and CXCL16. Western blotting showed decreased expression of p-PI3K, p-AKT, PCNA, MMP9, CXCL16, BCL-2 and BAX. Molecular docking predicted favorable binding to these targets. BD (0,0.7 mg/kg) reduced tumor volume by 87.4% without liver/kidney toxicity, and reduced Ki-67, PCNA, MMP9, CXCL16 in tumors.

**Conclusion:**

BD reduced prostate cancer growth and motility by inhibiting PI3K/AKT signaling and concomitantly downregulating CXCL16, and showed anti-tumor activity in a subcutaneous xenograft model with good safety. These results provide mechanistic information on skeletal metastasis in orthopedic oncology. Dedicated bone metastasis models need to be evaluated.

## 1 Introduction

Prostate cancer ranks among the most prevalent malignancies affecting men globally and constitutes a leading contributor to cancer-related mortality worldwide (Briganti et al., 2015). Although advances in early detection, surgical techniques, radiation therapy, and systemic interventions have extended patient survival, disease progression to skeletal metastasis precipitates substantial deterioration in quality of life. Patients experience debilitating osseous pain, pathological fractures, spinal cord compression, and hypercalcemia—complications that severely restrict physical mobility, diminish therapeutic tolerance, and elevate mortality rates (Kimura, 2018).

Androgen deprivation therapy remains the cornerstone of systemic management for advanced disease; however, this approach paradoxically accelerates bone mineral density loss and elevates fragility fracture risk. Ultimately, more than 80% of patients with late-stage prostate cancer develop skeletal metastases, which remain largely refractory to curative intervention (Suzman et al., 2014). Contemporary bone-targeted agents, including bisphosphonates, denosumab, and radium-223, are deployed to forestall or postpone skeletal-related events. While these therapies mitigate fracture incidence and alleviate osseous pain, they fail to eliminate established metastatic lesions and are associated with prolonged-use complications such as nephrotoxicity, medication-related osteonecrosis of the jaw, and additional adverse effects (Jara et al., 2022). This therapeutic inadequacy stems primarily from the predominant focus on suppressing osteoclastic bone resorption, while neglecting to comprehensively address the molecular mechanisms governing tumor cell homing to skeletal sites, microenvironmental adaptation, and subsequent proliferation within osseous niches (Sousa and Clézardin, 2018).

Bone metastasis is a multistep process (Coleman et al., 2020).Tumor cells first leave the primary site, then travel through the circulation, adhere to the bone marrow niche, and finally remodel the bone microenvironment through crosstalk with osteoclasts, osteoblasts, and stromal cells (Moschetta et al., 2017). Proteins like RANKL, chemokines, and integrins play a role in these steps. They guide the tumor cell migration, support survival in bone, and promote osteolytic or osteoblastic changes. Because there are many parallel pathways and interactions, agents that can hit several key nodes at the same time may offer better control of metastatic progression than single target drugs (Dougall and Chaisson, 2006).

Natural products and traditional medicines contain many compounds with multitarget low toxicity properties. They are now an important source of new antitumor drug discovery, especially in the field of metastasis. Bruceine D is a tetracyclic triterpenoid quassinoid isolated from the fruit of Brucea javanica (Yu et al., 2020). Previous studies showed that Bruceine D inhibits proliferation, induces apoptosis, and blocks invasion and migration in lung, breast, liver, pancreatic and colorectal cancer by regulating several signaling pathways (Mohan et al., 2021). Recent studies also suggest that Bruceine D may interfere with metastatic signaling (Zhu et al., 2023). For example, Bruceine D acts as a Trop2 inhibitor in breast cancer and can disrupt the Trop2/catenin positive feedback loop, reduce catenin nuclear translocation, reverse epithelial–mesenchymal transition and limit extracellular matrix remodeling (Tang et al., 2024). In addition, Bruceine D in combination with afatinib decreases DNA replication and clonogenic survival, arrests cell cycle arrest and apoptosis, which supports its potential as a chemosensitizer. Critically, existing studies have confirmed the efficacy of PI3K/AKT inhibitors in the treatment of advanced prostate cancer (Morgan et al., 2009).

CXCL16, a membrane-bound/soluble chemokine, drives prostate cancer progression via CXCR6 signaling, activating PI3K/AKT, ERK, NF-κB to promote proliferation, survival, EMT, and invasion (Ha et al., 2011). Tumor/stromal-derived CXCL16 also recruits immunosuppressive cells and upregulates MMP-9. Clinically, CXCL16 correlates with Gleason score, castration resistance, and poor prognosis, making it an ideal multi-target for advanced disease with skeletal tropism.

Despite increasing interest in natural products for metastatic cancer, research on BD in prostate cancer remains at an early stage and has largely focused on general anti-proliferative or pro-apoptotic effects. It is unclear how BD influences specific signaling networks that control tumor cell motility and interactions with microenvironmental cues linked to skeletal metastasis, such as the PI3K/AKT pathway and the chemokine CXCL16.

## 2 Materials and methods

### 2.1 Cell line selection rationale

PC-3 cells, established from a bone metastasis of prostate cancer (Kaighn et al., 1979), are androgen-independent (AR-negative), highly metastatic, PSA-negative, and exhibit neuroendocrine features, modeling castration-resistant bone-metastatic disease. C4-2B cells, derived from LNCaP xenografts via bone metastasis selection in nude mice (Garnero et al., 2000), maintain androgen receptor signaling despite castration resistance and demonstrate enhanced bone marrow homing and osteoblastic lesion formation. These lines collectively represent clinically relevant features of advanced, bone-involved prostate cancer progression (Spans et al., 2014).^,^ (Tong et al., 2015)

### 2.2 Cell culture

The human prostate cancer cell lines PC-3 and C4-2B were obtained from Wuhan Seville Biotechnology Co., Ltd. (Wuhan, China). PC-3 cells were grown in Ham’s F-12K medium (Gibco United States) with 10% FBS (Gibco Australia), 100 U/mL penicillin, 100 U/mL streptomycin (Gibco United States), C4-2B cells in RPMI-1640 medium (Gibco United States), 10% FBS, 100 U/mL penicillin and 100 U/mL streptomycin. Both cell lines were cultured at 37 °C in humidified atmosphere with 5% CO_2._

### 2.3 Rationale for concentration ranges in cell-based assays

The broad concentration range (0, 20, 40, 60, 80, 120, 140, 160, 180 μM) in the initial CCK-8 viability assay was selected to comprehensively establish dose-response curves and IC50 values across sub-toxic to cytotoxic levels. This approach aligns with studies of quassinoid compounds using 10–100 μM ranges to determine potency in prostate cancer cells (Lu et al., 2024).

Subsequent functional assays (migration, invasion, apoptosis: 0, 3.25,6.5,13, 26, 52 μM) employed a narrowed range bracketing the determined 24h IC50 values (PC-3: 15.8 ± 1.6 μM; C4-2B: 34.2 ± 2.1 μM), including sub-IC50, IC50-proximal, and moderate supra-IC50 concentrations. The highest concentration (52 μM) assesses maximal pathway modulation while pilot viability experiments confirmed >30% cell survival at 24h, ensuring sufficient metabolically active cells for motility and invasion assays. This refined range is consistent with published Brucein D studies using 2.5–20 μM (Lai et al., 2017) and structurally related Bruceine A studies employing 20–60 μM ranges (Zhang et al., 2023).

### 2.4 Cell counting kit-8 assay

Cells (3 × 10^3^/well) were seeded in 96-well plates and allowed to adhere overnight.Cells were exposed to BD at 0, 20, 40, 60, 80, 120, 140, 160, or 180 μM for 24, 48, or 72 h (vehicle: 0.2% DMSO). After treatment, 10 μL CCK-8 reagent (APExBIO, United States) was added per well, and cells were incubated for 2–4 h. Absorbance at 450 nm was measured using a microplate reader (Thermo Fisher Scientific, United States). Cell viability was calculated relative to vehicle controls. IC50 values were determined using GraphPad Prism 10.1.2: PC-3 = 15.8 ± 1.6 μM; C4-2B = 34.2 ± 2.1 μM

### 2.5 Colony formation assay

A total of 1 × 10^3^ cells were plated onto 6well plates and attached overnight. After 24 h cells were exposed to BD (0, 3.25, 6.5, 13, 26 or 52 μM (IC50 range PC-3: 15.8 ± 1.6 μM; C4-2B: 34.2 ± 2.1 μM) for 24 h, replaced by a complete medium. Colonies were fixed with 4% paraformaldehyde (Macklin) stained with 0.1% crystal violet for 20 min, washed, air dried and photographed after 14 days. Colonies were counted manually (24 h).

### 2.6 Cell migration assay

C4-2B and PC-3 cells were serum-starved overnight in RPMI-1640 + 0.5% FBS. 1 × 10^5^ cells in 200 μL serum-free medium containing BD (0, 3, 25, 6.5, 13, 26 or 52 μM) were seeded into the upper chambers of 24-well Transwell inserts (8 μm pores, Corning Inc., United States). The lower chambers contained 600 μL complete medium (10% FBS) as chemoattractant. After 24–48 h at 37 °C, non-invaded cells were removed from the upper surface, invaded cells were fixed in 4% paraformaldehyde (15 min), stained with 0.1% crystal violet (20 min), and counted in three random high-power fields per insert using ImageJ. Experiments were performed in triplicate.

### 2.7 Apoptosis assay (annexin V-FITC/PI staining)

Cells were seeded in 6-well plates, treated with BD (0, 3, 25, 6.5, 13, 26 or 52 μM) for 24 h, trypsinized, washed twice with cold PBS, and resuspended in 100 μL binding buffer. Cells were stained with 2.5 μL Annexin V-FITC and 2.5 μL PI (BD Biosciences, United States) for 15 min each at room temperature in the dark. Apoptosis was quantified by flow cytometry (FACS Aria III, BD Biosciences) gating early (Annexin V^+^/PI^−^) and late (Annexin V^+^/PI^+^) apoptotic cells.

### 2.8 Annexin-V/PI assay for apoptosis

Annexin V-FITC/propidium iodide (PI) staining facilitated apoptosis quantification via commercial detection kit (BD Biosciences, United States). Post 24-h Bruceine D exposure, adherent cells underwent trypsin detachment, followed by dual rinses with chilled PBS and resuspension in 100 μL 1× binding buffer. Annexin V-FITC reagent (2.5 μL) was introduced for 15-min room-temperature incubation shielded from light, then augmented with 400 μL binding buffer plus 2.5 μL PI prior to final 15-min dark incubation. Flow cytometric evaluation employed FACS Aria III sorter (BD Biosciences, United States), stratifying early apoptosis (Annexin V-FITC^+^/PI^−^) from late apoptosis/necrosis (Annexin V-FITC^+^/PI^+^) based on relative proportions within gated live cell populations.

### 2.9 Transwell invasion assay

C4-2B and PC-3 prostate cancer cell lines underwent overnight serum deprivation in RPMI-1640 supplemented with 0.5% FBS to achieve cell cycle synchronization while minimizing proliferative activity. Transwell invasion assays employed 24-well inserts equipped with 8-μm pore-size polycarbonate filters (Corning Inc., New York, United States). Upper chambers received 1 × 10^5^ cells suspended in 200 μL serum-free medium containing BD (0, 3.25,6.5, 13, 26, 52 μM), while lower chambers contained 600 μL complete medium with 10% FBS functioning as chemoattractant. After 24–48 h incubation under standard conditions (37 °C, 5% CO_2_), non-migrated cells on the upper membrane surface were carefully swabbed away. Cells penetrating to the lower surface underwent fixation in 4% paraformaldehyde (15 min), followed by 0.1% crystal violet staining (20 min, room temperature), and subsequent microscopic documentation. Per insert, three randomly selected high-power fields were photographed via inverted phase-contrast microscopy, with invaded cell enumeration performed using ImageJ analysis software. Each condition incorporated three independent biological replicates; quantitative results appear as mean ± SD with statistical comparisons via unpaired t-tests or one-way ANOVA where appropriate.

### 2.10 Western blot

Tissue samples and cultured cells received graded Bruceine D (BD) exposures lasting 24 h. Post-incubation, BD-supplemented medium underwent aspiration, followed by single gentle rinse of samples using pre-chilled PBS. Cellular lysates were generated through ice incubation (30 min) in 200 μL RIPA buffer fortified with 1% PMSF alongside protease/phosphatase inhibitor cocktails, subjected thereafter to 12,000 × g centrifugation (10 min, 4 °C) yielding clarified supernatants. Total protein content within supernatants underwent BCA quantification (Thermo Fisher Scientific, United States).Normalized protein loads (20–40 μg/lane) received Laemmli buffer denaturation via 5-min boiling. SDS-PAGE resolution occurred across 8%–12% gradient gels (100 V, 90 min), followed by electrophoretic transfer to methanol-activated PVDF membranes (wet tank; 300mA, 90 min, 4 °C). The membranes were blocked with 5% non-fat milk in TBST, followed by overnight incubation at 4 °C with primary antibodies at manufacturer-recommended dilutions: PI3K (AF5112, Affinity Biosciences), phospho-PI3K (AF6241, Affinity Biosciences), AKT (AF6261, Affinity Biosciences), phospho-AKT (AF3263, Affinity Biosciences), PCNA (10205-2-AP, Proteintech), MMP9 (10375-2-AP, Proteintech), CXCL16 (60123-1-Ig, Proteintech), BAX (AF0120, Affinity Biosciences), BCL2 (AF6139, Affinity Biosciences), GAPDH (AF7021, Affinity Biosciences), and Tubulin (AF7011, Affinity Biosciences). Triple 15-min TBST washes preceded 1-h HRP-conjugated secondary antibody (goat anti-rabbit IgG) hybridization at ambient temperature, succeeded by three additional TBST rinses (15 min each). Enhanced chemiluminescence (ECL) development facilitated band visualization, with densitometric quantification via Tanon-5200 chemiluminescence imager. Quantification: ImageJ analysis (1) Raw bands → GAPDH-normalized; (2) p-AKT/total AKT and p-PI3K/total PI3K ratios calculated from GAPDH-normalized values. CV of GAPDH: <12% across lanes.

### 2.11 RNA extraction and RNA sequencing analysis

1 × 10^6^ PC-3 and C4-2B cells were randomly assigned to control and Bruceine D (BD)-treated groups (n = 3 biological replicates/group), with BD-treated cells receiving 26 μM BD for 24h. **Total RNA was extracted** from each sample using TRIzol reagent (Invitrogen, United States) per manufacturer’s protocol, yielding ≥1 μg total RNA (A260/280 = 1.8–2.0). Samples were submitted to Omicsmart (Guangzhou, China) for high-throughput RNA sequencing analysis.

### 2.12 Gene set enrichment analysis (GSEA)

Transcriptome sequencing data underwent bioinformatic processing via the Dynamic GSEA enrichment analysis tool within the OmicShare platform (http://www.omicsmart.com/). Analysis employed the *Homo sapiens* reference genome assembly (GRCh38.p13). Key pathways modulated by Bruceine D (BD) in prostate cancer were identified through MSigDB gene set collections. Differential expression analysis was complemented by Gene Ontology (GO) enrichment and Kyoto Encyclopedia of Genes and Genomes (KEGG) pathway analyses.

### 2.13 Molecular docking

Molecule docking is a predictive technique that allows small molecules to be bound to protein receptors. We used semi-flexible docking to get a more stable complex conformation needed for understanding the action of small molecules and for screening lead compounds. The three-dimensional structure of the ligand Bruceine D was obtained from PubChem Compound (https://pubchem.ncbi.nlm.nih.gov/#query=Bruceine+D). The receptor proteins AKT, PIK3AP1, PIK3CD, PIK3R2, and CXCL16 were retrieved from the RCSB Protein Data Bank (https://www.rcsb.org/).We docked Bruceine D (PubChem CID: 441788) and the following protein targets: AKT1 (PDB ID: Interface with 7FCV), PIK3CD (PDB ID: 8BCY), PIK3R2 (PDB ID: 2KT1), PIK3AP1 (PDB ID: 5FOR) and CXCL16 (PDB ID: AF-Q9H2A7-F1). Small molecules ligands and proteins are pretreated before docking. For proteins we use PyMOL 3.0.3 to remove water molecules, irrelevant ligands and adding hydrogen. Compounds are energy minimization with ChemDraw 20.0. Then we generated PDBQT file with AutoDock Tools 1.57 where mesh box of the whole protein is set to %C% while default parameters remain intact. Molecular docking simulations generated nine binding conformations per ligand-receptor pair. Conformations demonstrating optimal binding affinity (lowest free energy) alongside maximal clustering occupancy served as representative ligand-receptor docking poses. Docking results were subsequently visualized using PyMOL version 3.0.3 to elucidate molecular interactions and binding geometry between Bruceine D and target proteins. Two-dimensional interaction diagrams were generated using Discovery Studio Visualizer.

### 2.14 Animal treatment with bruecine D

Twenty-two 5-week-old male BALB/c nude mice were procured from Beijing Weitonglihua Laboratory Animal Technology Co., Ltd. All animal procedures adhered to the National Institutes of Health “Guide for the Care and Use of Laboratory Animals” (NIH Publication No. 8023, revised 1978) and received approval from the Institutional Animal Care and Use Committee of Wuhan Selway Biological Technology Co., Ltd. Tumor-bearing mice were monitored until mean tumor volume reached approximately 100 mm^3^, at which point randomization occurred based on individual tumor size to ensure balanced group allocation. Baseline body weights and tumor dimensions (measured using digital calipers) were recorded prior to treatment initiation. Animals were stratified into three experimental groups (n = 6–7 per group): vehicle control (5% DMSO administered intraperitoneally), low-dose Bruceine D (0.2 mg/kg intraperitoneally), or high-dose Bruceine D (0.7 mg/kg intraperitoneally).Doses of 0.2 and 0.7 mg/kg were selected based on preliminary maximum tolerated dose studies in our laboratory and published data on structurally related quassinoids. Tong et al. (2015) demonstrated that 5–10 mg/kg of a standardized quassinoid composition from Eurycoma longifolia effectively suppressed LNCaP prostate cancer xenograft growth without significant toxicity (Tong et al., 2015). Lu et al. (2024) reported comparable antitumor efficacy of bruceine A at 0.5 mg/kg to gemcitabine at 25 mg/kg (Lu et al., 2024). These findings support the tolerability and efficacy of quassinoid compounds in the low mg/kg range in xenograft models, justifying our exploratory low (0.2 mg/kg) and high (0.7 mg/kg) dose regimen. Treatment continued for 21 consecutive days. At study endpoint, mice were euthanized with pentobarbital anesthesia, and terminal blood samples (100–200 μL) were collected from the retro-orbital sinus using heparinized microcapillary tubes. Plasma was isolated by centrifugation at 1,200 × g for 15 min at 4 °C. Tumors and major organs (liver, kidney, heart, spleen) were excised, weighed, and tumor volumes calculated using the formula: TV = (π/6) × L × W^2^, where L represents the longest axis and W the perpendicular short axis dimension. The study protocol was approved by Beijing Vital River Laboratory Animal Technology Co., Ltd. (Approval No.: No.110011251109382535, License No.: SCXK(Beijing) 2021 - 0006).Hematoxylin and Eosin staining

Tissue specimens were fixed in 4% paraformaldehyde, processed, and embedded in paraffin. Serial 5-μm sections were cut from paraffin blocks and subjected to hematoxylin and eosin (H&E) staining following standard histopathological protocols. Stained sections were examined and imaged using a Nikon Eclipse light microscope (Nikon Instruments, Japan).

### 2.15 Serum biochemical tests

Serum concentrations of alanine aminotransferase (ALT), aspartate aminotransferase (AST), and creatinine (Cr) were quantified using commercial assay kits from Radu Life Sciences Co., Ltd. according to manufacturer instructions.

### 2.16 Statistical analysis

Quantitative results appear as mean ± standard deviation (SD). Intergroup comparisons utilized one-way ANOVA, deeming *p < 0.05, **p < 0.01, and ***p < 0.001 indicative of statistical significance.

## 3 Results

### 3.1 Subsection

#### 3.1.1 Bruceine D-mediated suppression of human prostate cancer cell proliferation

**FIGURE 1:**
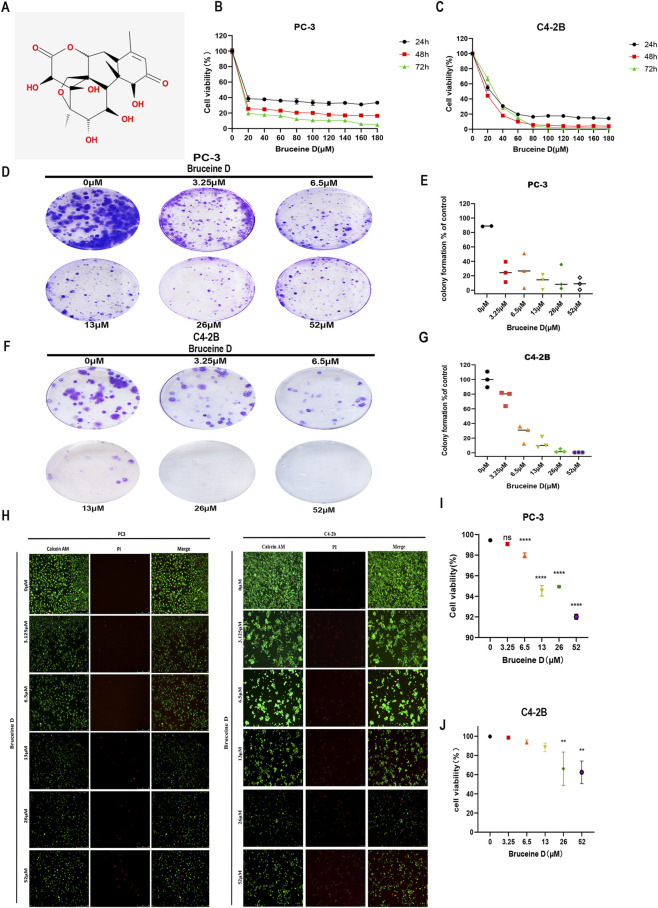
Effects of BD on the proliferation of human prostate cancer cells. **(A)** Chemical structure of BD **(B,C)** Changes in viability of PC-3 and C4-2B prostate cancer cells after 24, 48, and 72 h of BD treatment (Vehicle (0.2% DMSO) at 0 μM; all BD concentrations normalized to equivalent 0.2% DMSO). **(D,F)** Representative colony formation of PC-3 and C4-2b cells following 24h exposure to 0, 3.25,6.5,13, 26, 52 μM BD. **(E,G)** Manual counts of colonies formed in each treatment group. **(H)** Live-to-dead cell ratios in PC-3 and C4-2B cultures after 24 h of BD treatment at different concentrations. **(I,J)** Quantification of fluorescence intensities for live and dead cell staining. Statistical significance was determined by one-way analysis of variance and is indicated as *p < 0.05, **p < 0.01, ***p < 0.001 versus the control.

**FIGURE 2:**
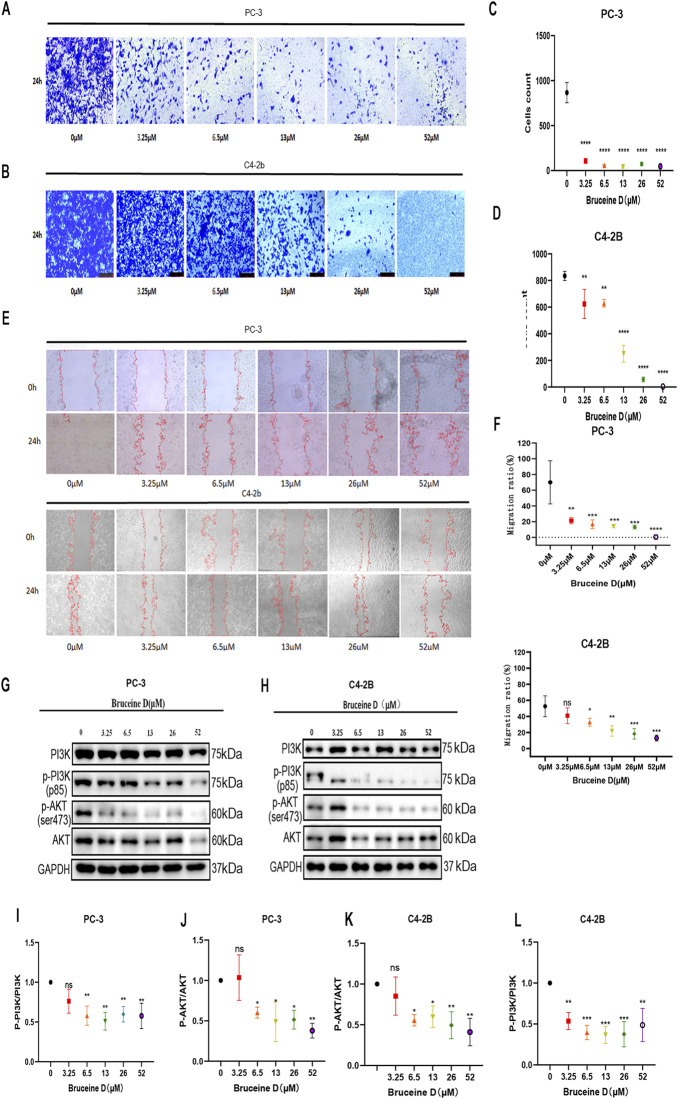
BD inhibits migration and motility of prostate cancer cells. **(A–D)** Distribution of prostate cancer cell lines (PC-3 and C4-2b) after passage through the chamber following 24-h treatment with varying doses of BD. **(E,F)** Wound-healing analysis of PC-3 and C4-2B cells 24 h after BD treatment, performed by the scratch assay. **(G-L)** Western blot analysis of P-PI3K and P-AKT expression in PC-3 and C4-2b cells after 24-h BD treatment. Data are presented as mean ± standard deviation from three independent experiments. One-way ANOVA was used to compare each group with the control. *p < 0.05, **p < 0.01, and ***p < 0.001 denote statistical significance.

The chemical structure of Bruceine D is shown in Figure 1A. Cell viability after BD exposure was measured using CCK-8 at 0–180 μM(20 μM increments) for 24, 48, 72h. BD reduced viability time- and dose-dependently (Figures 1B,C). 24h IC50 values: PC-3 = 15.8 ± 1.6 μM; C4-2B = 34.2 ± 2.1 μM (Figures 1B,C, GraphPad Prism 9.0). PC-3 cells exhibited greater sensitivity than C4-2B, with ∼2.2-fold lower IC50 and steeper viability decline at 20–60 μM. Colony formation confirmed cytostatic effects (Figures 1D–G). Western blotting showed BD suppressed p-PI3K/PI3K and p-AKT/AKT ratios (Figures 2G,H). Calcein-AM/PI staining revealed dose-dependent live cell reduction and dead cell increase (Figures 1H,I), confirming BD’s concentration-dependent antiproliferative and cytotoxic activity.

#### 3.1.2 Bruceine D suppression of human prostate cancer cell migration and invasion

**FIGURE 3:**
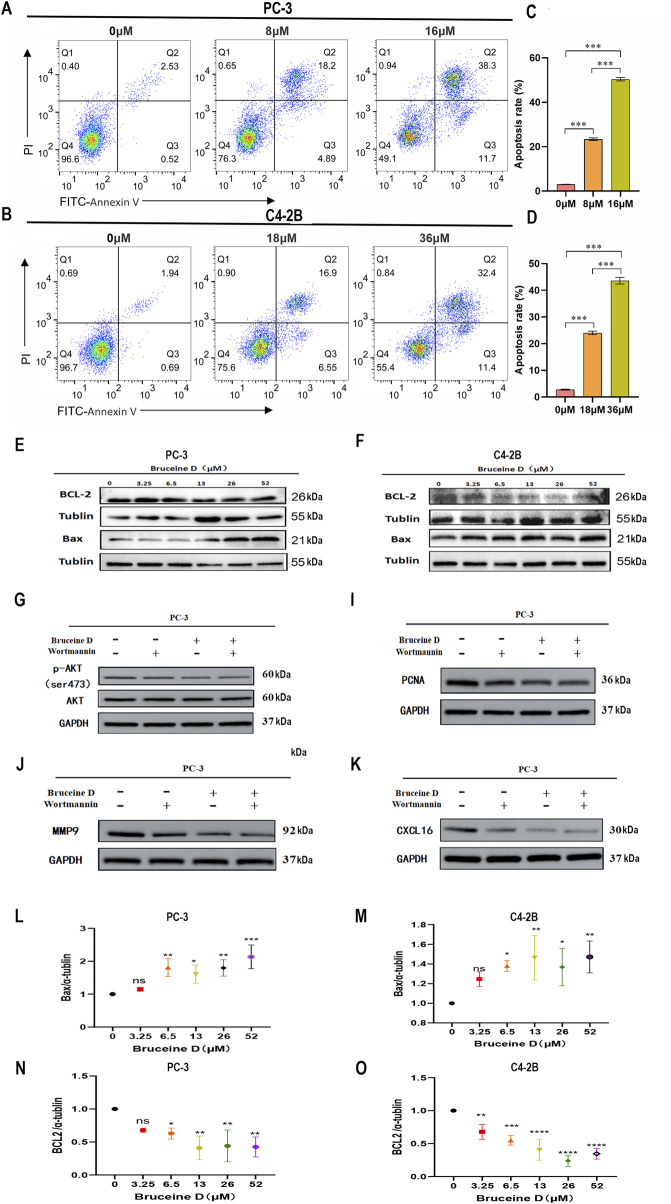
BD-induced apoptosis in prostate cancer cells. **(A–D)** Annexin V-FITC/PI staining quantified apoptotic fractions in PC-3 and C4-2b cells exposed to varying BD concentrations. **(E,F)** After 24 h of BD treatment, Western blotting assessed BAX and Bcl-2 expression. **(G-N)** Western blotting evaluated P-AKT, PCNA,MMP9,CXCL16 and related proteins in control, positive control, and BD-treated groups to reveal protein-level changes and relationships. Data are presented as mean ± S.D. from three independent experiments. Statistical significance was assessed by one-way ANOVA; *p < 0.05, **p < 0.01, and ***p < 0.001 versus the control.

In the Transwell invasion assay, increasing concentrations of Bruceine D reduced the number of PC-3 and C4-2B cells that penetrated the Matrigel-coated membrane and appeared on the lower surface of the Transwell insert (Figures 2A–D), indicating a concentration-dependent inhibition of invasion.All these results show that Bruceine D inhibits migratory and invasive phenotypes of prostate cancer cells. To better evaluate the apoptotic effects of BD, Annexin V-FITC/PI flow cytometry was performed using concentrations below the IC50 values specific to each cell line. PC-3 cells were treated with 0, 8, and 16 μM BD (IC50 = 15.8 μM), while C4-2B cells were treated with 0, 18, and 36 μM BD (IC50 = 34.2 μM). Under these revised conditions, BD induced a concentration-dependent increase in total apoptotic rates in both cell lines (P < 0.01), with trends consistent with the CCK-8 viability results (Figures 3A–D).Importantly, at lower concentrations, treated cells predominantly exhibited early apoptosis (Annexin V+/PI-), characterized by progressive phosphatidylserine externalization with intact cell membranes. At higher concentrations approaching IC50, a shift toward late apoptosis (Annexin V+/PI+) was observed, indicating loss of membrane integrity. Immunoblotting showed mechanistic evidence. Immunoblotting showed mechanistic evidence of Bruceine D-mediated upregulation of pro-apoptotic Bax with downregulation of anti-apoptotic Bcl-2 (Figures 3E,F). These results suggest Bruceine D is a potent apoptosis inducer which compromises prostate cancer cell survival in PC-3 and C4-2B models.

#### 3.1.3 BD modulates the PI3K/AKT pathway and downregulates CXCL16 expression

**FIGURE 4:**
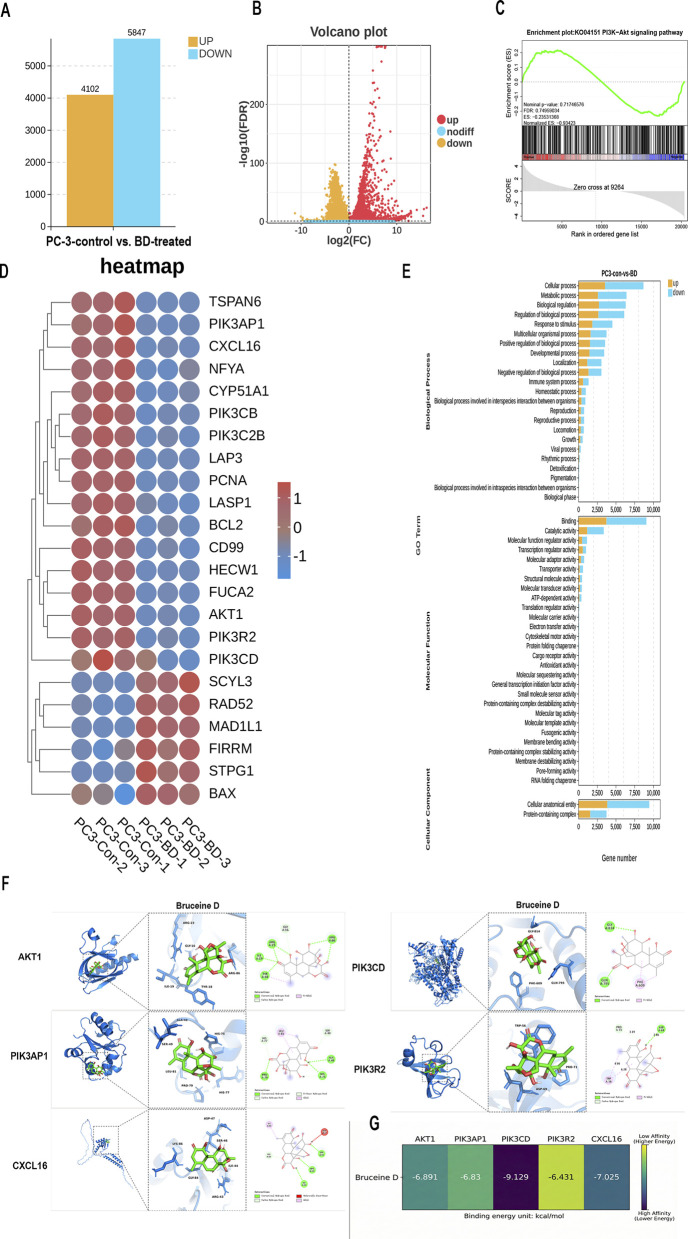
Effects of BD on the PI3K/AKT pathway and the CXCL16 chemokine. **(A,B,D)** Bar plots, volcano plots, and heatmaps depict differentially expressed genes in PC-3 cells treated with BD for 24 h (n = 3). **(C)** GSEA enrichment plot for the PI3K/AKT pathway gene set. **(E)** GO enrichment analysis of biological processes (BP), cellular components (CC), and molecular functions (MF). **(F,G)** Molecular docking models of ligand BD with the protein receptors PIK3AP1, PIK3C2B, PIK3CB, PIK3R2, AKT1, and CXCL16.Statistical significance was determined by one-way analysis of variance and is indicated as *p < 0.05, **p < 0.01, and ***p < 0.001 versus the control.

We sequenced PC-3 cells from the control group and cells from the group treated with 26 μM Bruceine D for differentially expressed genes. Treatment with Bruceine D changed expression of 9,949 genes (5,847 downregulated and 4,102 upregulated).Among differentially expressed genes, PIK3AP1 had log2FC of −3.01 (FDR 0), PIK3C2B log2FC of 3.58 (FDR 0), PIK3CB log2FC of −2.23 (FDR 0), PIK3R2 log2FC of −1.85 (FDR 0), AKT1 log2FC of 3.79 (FDR 0) and CXCL16 log2FC of −3.07 (FDR 0).These results show that the above genes were downregulated by about 6, 7, 4.4, 3.7, 7.6, and 6 times (FDR 0) after Bruceine D treatment, and differences were statistically significant. These findings suggest that CXCL16 and PI3K/AKT pathway components represent candidate targets of Bruceine D action in prostate cancer cells. To determine the source of CXCL16 in our model, we first confirmed its endogenous expression in the studied prostate cancer cell lines. Transcriptomic profiling of PC-3 cells revealed high baseline expression of CXCL16, which was significantly downregulated (log_2FC = −3.07, FDR = 0) following Bruceine D treatment. Consistent with these mRNA findings, protein analysis via Western blotting confirmed that both PC-3 and C4-2B cells constitutively express CXCL16, and this expression was reduced by BD in a dose-dependent manner. Furthermore, immunohistochemical staining of subcutaneous xenograft tissues demonstrated that CXCL16 is localized within the tumor cells themselves, confirming that the cancer cells are a primary source of this chemokine in our model. These data suggest that BD disrupts a tumor-cell-derived CXCL16 signaling axis. Previous work shows that lower binding energy between a receptor and its ligand increases affinity and conformational stability. Protein analysis showed dose-dependent decreases in phosphorylated PI3K, AKT, and CXCL16 levels following BD treatment, consistent with the known effects of PI3K/AKT pathway inhibition (as seen with Wortmannin) (Figures 3G–L).Levels of downstream proteins associated with extracellular matrix degradation (MMP9) and cell cycle progression (PCNA) were also decreased. Together, these data indicate that BD is associated with downregulation of PI3K/AKT-related signaling and CXCL16, as evidenced by functional suppression of proliferation, migration, and invasion. These findings suggest a possible mechanistic association rather than a formally validated linear PI3K/AKT-CXCL16-MMP9 cascade (Figure 4).

#### 3.1.4 Bruceine D suppression of prostate tumor xenograft growth *in vivo*

**FIGURE 5:**
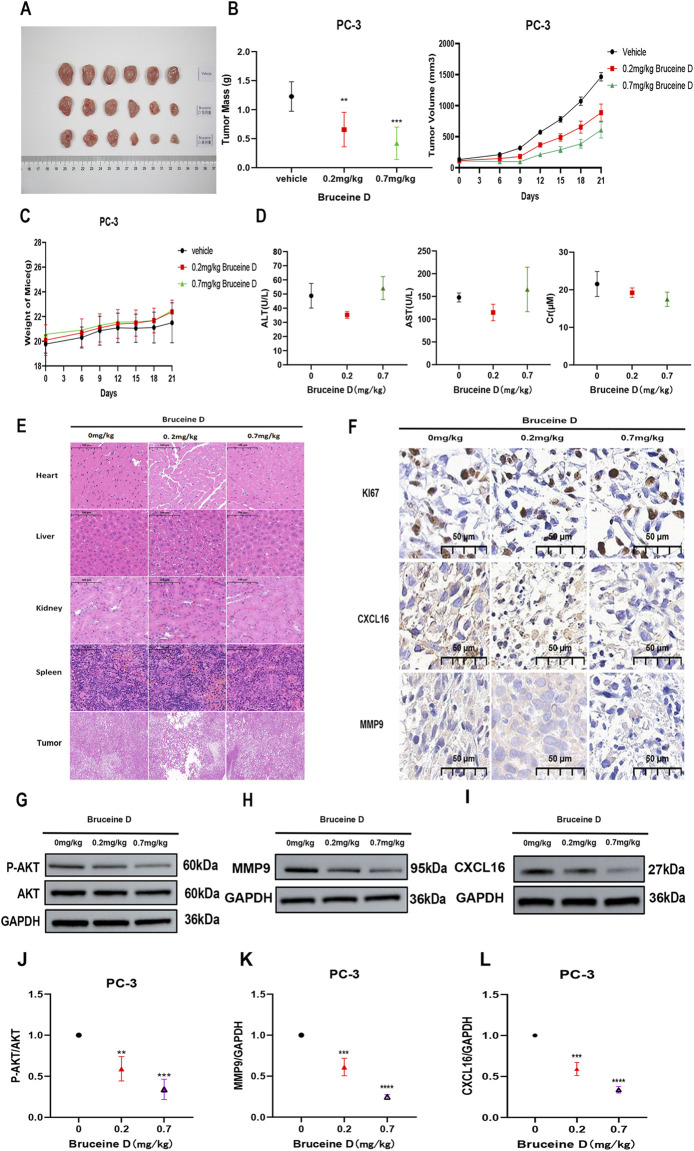
Effect of BD on the growth of subcutaneous anterior tumors in mice. **(A)** Representative images of tumors from mice treated with different BD concentrations for 21 days (n = 6). **(B)** Tumor weight and tumor volume in control and BD-treated groups. **(C)** Body weight changes of tumor-bearing mice during treatment. **(D)** Changes in serum ALT, AST, and Cr levels. **(E)** H&E-stained images of heart, liver, kidney, spleen, and tumor tissues from mice (n = 5). **(F)** Immunohistochemical staining for Ki-67, CXCL16, and MMP9 in tumor tissues (n = 5). **(G–L)** Changes in P-AKT, CXCL16, and MMP9 expression in animal tissues after BD treatment assessed by Western blot, with GAPDH as the loading control. Statistical significance was determined by one-way analysis of variance and is indicated as *p < 0.05, **p < 0.01, ***p < 0.001 versus the control.

In comparison with the control group, Bruceine D treatment significantly decreased the tumor volume and body weight (Figures 5A,B), body weight did not change significantly over the 21 days treatment period (Figure 5C). Serum biochemical analysis showed that the ALT, AST and Cr levels were much better controlled in Bruceine D group (Figure 5D).Histopathological analysis of heart, liver, spleen, and kidney tissues revealed negligible post-treatment alterations. H&E staining revealed dose-dependent tumor changes (Figure 5E). Controls showed densely packed viable cells with frequent mitoses.0.2 mg/kg BD reduced cell density with increased extracellular matrix (ECM).0.7 mg/kg BD exhibited extensive ECM, sparse pyknotic cells, and absent mitosesImmunohistochemical assessment of proliferation markers Ki-67 and PCNA revealed substantial reductions in proliferative activity within Bruceine D-treated tumors. Likewise, MMP9 and CXCL16 immunostaining demonstrated marked suppression of tumor cell invasiveness and metastatic capacity in the treatment cohort (Figure 5F). Western blot analysis showed that Bruceine D suppressed AKT and CXCL16 protein expression in transplant mouse tumors (Figures 5G–L). Together, these results show that Bruceine D effectively inhibits prostate cancer *in vitro* without toxicity to liver or kidneys.

## 4 Discussion

In this study, we show that Bruceine D exerts potent antiproliferative, pro-apoptotic, and anti-motility effects in prostate cancer cells and suppresses tumor growth *in vivo*, while being associated with inhibition of the PI3KAKT pathway and downregulation of CXCL16 and MMP9.These results provide mechanistic insight into how a multitarget natural product can disrupt pathways associated with aggressive, bone-tropic prostate cancer and establish a pharmacological rationale for further evaluation of BD in orthopedic oncology.

Our data show that BD markedly suppresses prostate cancer cell growth and motility by inhibiting PI3K/AKT signaling, with concurrent reduction of CXCL16 and MMP9 expression. In PC-3 and C4-2B cells, BD reduced viability and clonogenic survival in a time- and concentration-dependent manner and impaired wound closure and Transwell invasion, indicating broad suppression of proliferative and migratory capacity. At the molecular level, BD lowered PI3K and AKT phosphorylation and reduced expression of the proliferation marker PCNA and the matrix-degrading enzyme MMP9, consistent with inhibition of a central survival and motility pathway. Concurrent upregulation of the pro-apoptotic protein BAX and downregulation of the anti-apoptotic protein BCL2 indicate that BD shifts the balance toward intrinsic apoptosis downstream of PI3K/AKT inhibition. These results align with prior reports linking PI3K/AKT hyperactivation to aggressive, treatment-resistant prostate cancer and support the view that pathway inhibition can limit proliferation and invasion (He et al., 2021; Hashemi et al., 2023). Notably, BD appears to act on multiple nodes within this network rather than on a single catalytic subunit; classical PI3K/AKT inhibitors (Jia et al., 2023) often target one component and can be susceptible to compensatory escape signaling. This network-level mode of action may explain how BD simultaneously restrains growth, motility, and survival in prostate cancer cells. The stronger growth inhibition observed in PC3 cells than in C42B cells may reflect differences in baseline PI3K/AKT activity or genetic background and warrants further investigation.

Our findings are consistent with reports that BD suppresses proliferation, migration, and invasion while promoting apoptosis in lung, breast, liver, pancreatic, and colorectal cancers (Fan et al., 2020; Luo et al., 2020; Cheng et al., 2017; Liu et al., 2012; Yang et al., 2024), which supports its role as a multitarget natural product with broad anticancer potential. In breast cancer, BD has been reported to disrupt metastatic signaling and epithelial–mesenchymal transition–related processes (Li et al., 2018). Extending these observations, our study links BD’s antitumor effects to a prostate cancer–specific pathway tied to skeletal tropism. In this context, the downregulation of CXCL16 is especially pertinent because the CXCL16–CXCR6 axis has been shown to drive directional migration and bone marrow homing of prostate cancer cells (Ha et al., 2011).Our transcriptomic and proteomic data show that BD reduces CXCL16 expression in PC-3 cells and in xenograft tissues, with a concurrent decrease in MMP9 levels. Because MMP9 is a principal mediator of extracellular matrix degradation, these changes align with diminished invasive capacity and imply that BD may suppress chemokine-associated invasive signaling. Thus, BD could concurrently disrupt survival and motility signaling and attenuate CXCL16 and MMP9 expression, suggesting a coordinated regulatory network related to PI3K/AKT inhibition. However, because our functional assays used a two-dimensional culture system and a subcutaneous xenograft model, we cannot conclude that BD blocks skeletal homing or colonization *in vivo*. Orthotopic and intratibial studies are therefore required to determine whether this network governs *bona fide* bone metastasis within bone-specific microenvironments.

Previous studies have reported that the CXCL16/CXCR6 axis can activate PI3K/AKT signaling and promote MMP expression in prostate cancer. Therefore, our current data should not be interpreted as proving a strict upstream-downstream relationship among PI3K/AKT, CXCL16, and MMP9. Instead, our findings indicate that BD suppresses PI3K/AKT phosphorylation while concomitantly reducing CXCL16 and MMP9 expression, suggesting disruption of a functionally connected regulatory network.

The *in vivo* data further supports BD’s antitumor activity. In a subcutaneous xenograft model, BD markedly reduced tumor burden and lowered Ki67, PCNA, MMP9, and CXCL16 expression without detectable hepatic or renal toxicity, indicating a favorable initial therapeutic window. These findings are promising for orthopedic oncology, where systemic agents that curb tumor growth while preserving organ function are needed for patients with skeletal disease. However, the subcutaneous site does not reproduce the complex bone marrow microenvironment, which includes osteoclasts, osteoblasts, mesenchymal stem cells, and immune cells that critically influence metastatic colonization and tumor outgrowth (Chen et al., 2021). Thus, our current *in vivo* results demonstrate antitumor efficacy and preliminary safety but do not establish that BD can prevent or treat established bone metastases.

This study has several limitations. First, all *in vivo* experiments used a subcutaneous xenograft model, which does not reproduce the bone microenvironment; therefore, our findings provide mechanistic insight relevant to skeletal metastases but do not directly assess BD activity in bone lesions. Second, the PI3K/AKT–CXCL16 axis was inferred from transcriptomics, Western blotting, and *in silico* docking, but direct target engagement was not validated by biophysical methods such as surface plasmon resonance or CETSA. Third, we did not assess BD effects on osteoclasts, osteoblasts, bone marrow stromal cells, or immune cells, all of which are central to skeletal remodeling and metastatic colonization. Future studies employing orthotopic and intratibial metastasis models, combined with biophysical target validation and evaluation of BD effects on bone-resident cells, will be essential to define BD’s therapeutic potential for skeletal metastasis in orthopedic oncology.

**FIGURE 6:**
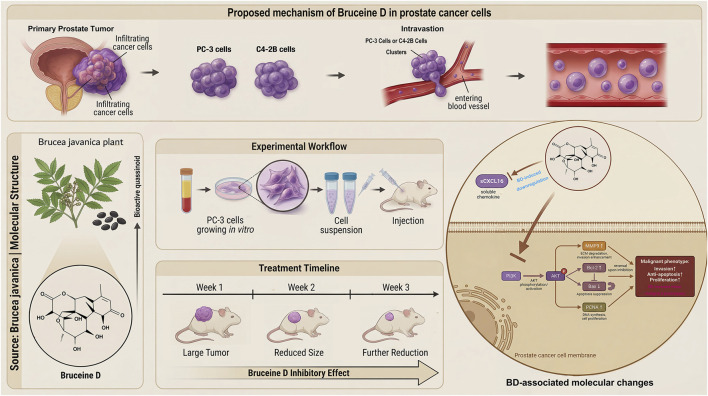
BD suppresses PI3K/AKT, CXCL16, and MMP9.

## 5 Conclusion

In summary, Bruceine D exerts significant antitumor effects in prostate cancer cells, including inhibition of proliferation, migration, and invasion, together with induction of apoptosis. These effects are associated with suppression of PI3K/AKT signaling and concomitant downregulation of CXCL16 and MMP9. Our findings suggest that BD may disrupt a coordinated regulatory network involved in tumor growth and motility rather than a formally validated linear PI3K/AKT-CXCL16-MMP9 cascade. Although further studies are needed to define the precise directionality and causal relationships within this network, our results provide preliminary mechanistic evidence supporting the potential of BD as a multitarget candidate for prostate cancer intervention (Figure 6).

## 6 Simple Summary

Skeletal metastasis is a major clinical challenge in advanced prostate cancer in orthopedics. Bruceine D (BD), a natural compound from Brucea javanica, inhibits proliferation, migration, invasion, and promotes apoptosis in PC-3 and C4-2B prostate cancer cells by suppressing PI3K/AKT signaling and concomitantly downregulating CXCL16. In subcu- taneous xenograft models, BD significantly reduces tumor burden without evidence of hepatotoxicity or nephrotoxicity. These results suggest that BD is a promising multi-target agent for controlling skeletal metastasis and warrant further evaluation in bone metastasis models.

## Data Availability

The original contributions presented in the study are publicly available. This data can be found here: https://www.ncbi.nlm.nih.gov/sra/PRJNA1469481; https://www.ncbi.nlm.nih.gov/sra/PRJNA1470923.
