# Supplementary material for: Bruceine D attenuates prostate cancer growth and motility by suppressing PI3K/AKT signaling and downregulating CXCL16: implications for skeletal metastasis in orthopedic oncology
Source: Front Pharmacol. 2026 Jun 1;17:1819854. doi: 10.3389/fphar.2026.1819854 (PMC13266102; doi:10.3389/fphar.2026.1819854)
Supplement: Supplementary file 1 [file DataSheet1.zip › Supplementary Materials/1.The specific antibody catalog number and dilution of WB.docx]

**TableS1 .** The specific antibody catalog number and dilution of WB.

| **Protein name** | **Brand** | **Cat No.** | **Dilution ratio** |
| --- | --- | --- | --- |
| PI3K * | Affinity | AF5112 | 1：1000 |
| P-PI3K | Affinity | AF6241 | 1：1000 |
| AKT | Affinity | AF6261 | 1：1000 |
| P-AKT | Affinity | AF3263 | 1：1000 |
|  |  |  |  |

| **Protein name** | **Brand** | **Cat No.** | **Dilution ratio** |
| --- | --- | --- | --- |
| PCNA * | Proteintech | **10205-2-AP** | 1：5000 |
| MMP9 | Proteintech | **10375-2-AP** | 1：1000 |
| CXCL16 | Proteintech | **60123-1-Ig** | 1：1000 |
| BAX | Affinity | AF0120 | 1：1000 |
| BCL2 | Affinity | AF6139 | 1：1000 |

| **Protein name** | **Brand** | **Cat No.** | **Dilution ratio** |
| --- | --- | --- | --- |
| GAPDH | Affinity | AF7021 | 1：1000 |
| Tubulin | Affinity | AF7011 | 1：1000 |
|  |  |  |  |
