# Supplementary material for: Bruceine D attenuates prostate cancer growth and motility by suppressing PI3K/AKT signaling and downregulating CXCL16: implications for skeletal metastasis in orthopedic oncology
Source: Front Pharmacol. 2026 Jun 1;17:1819854. doi: 10.3389/fphar.2026.1819854 (PMC13266102; doi:10.3389/fphar.2026.1819854)

Supplementary Material

# Supplementary Data

Supplementary Material should be uploaded separately on submission. Please include any supplementary data, figures and/or tables.

Supplementary material is not typeset so please ensure that all information is clearly presented, the appropriate caption is included in the file and not in the manuscript, and that the style conforms to the rest of the article.

# Supplementary Figures and Tables

For more information on Supplementary Material and for details on the different file types accepted, please see [here](https://www.frontiersin.org/guidelines/author-guidelines#supplementary-material).

## Supplementary Figures

**Supplementary TableS1 . The specific antibody catalog number and dilution of WB.**


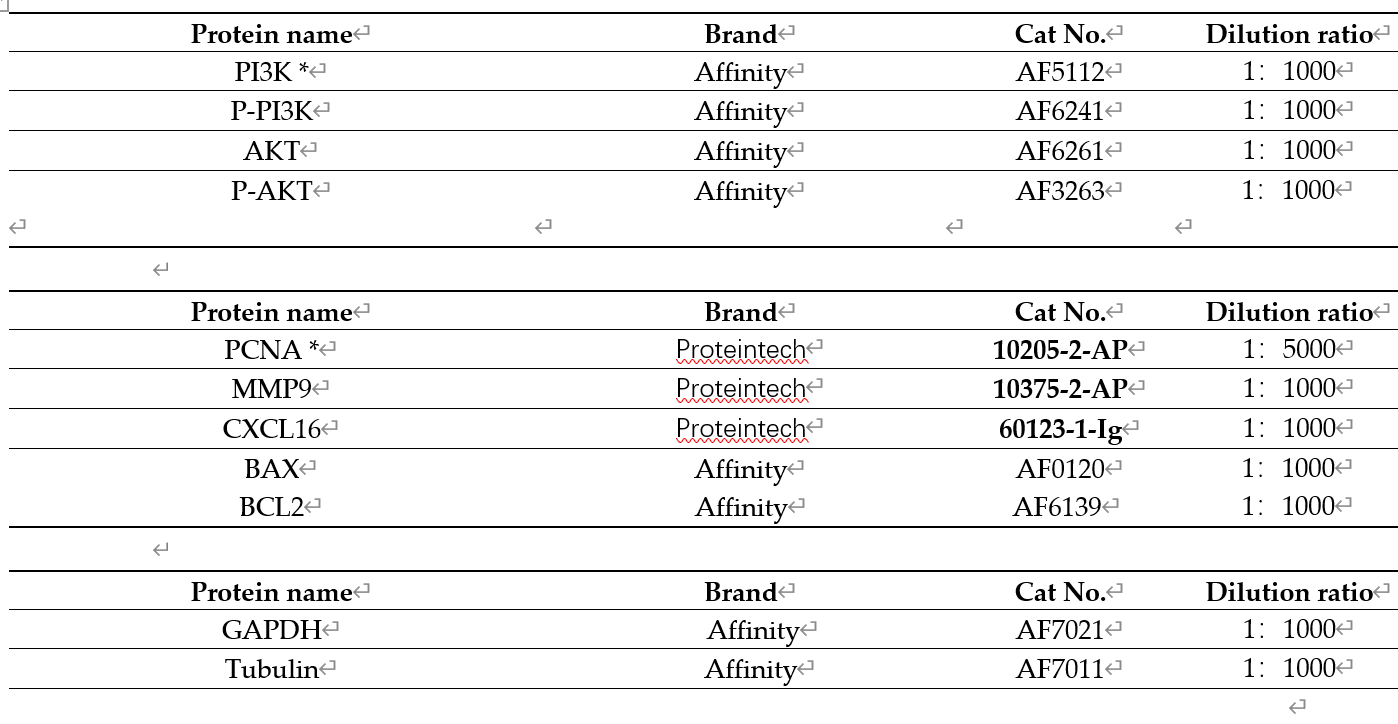


**Supplementary TableS2 . The binding energy of ligand Bruceine D to the receptor below。**
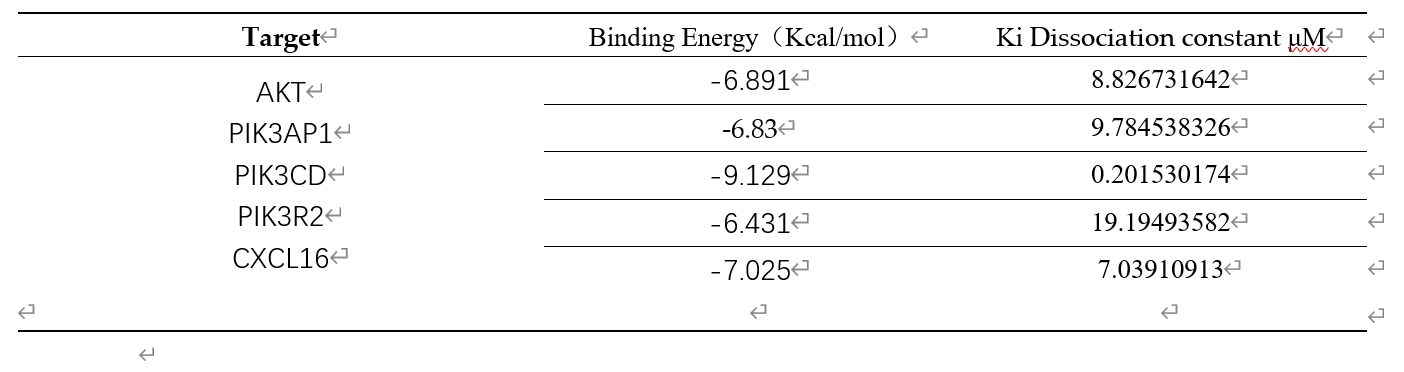


**Supplementary Figure1.Animal sampling and photography procedures are detailed in Supplementary Methods.**


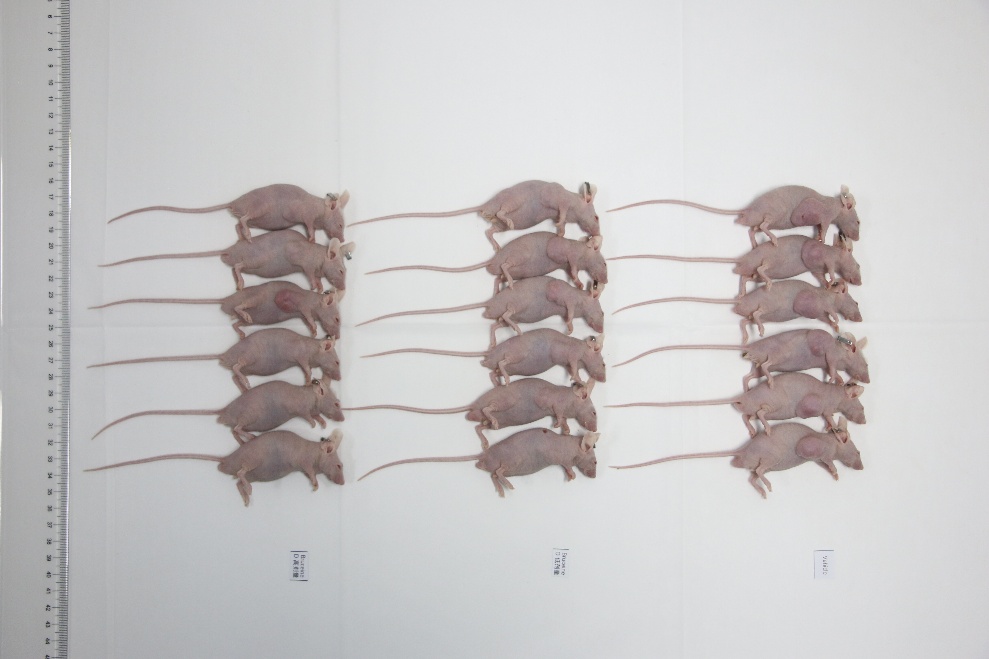

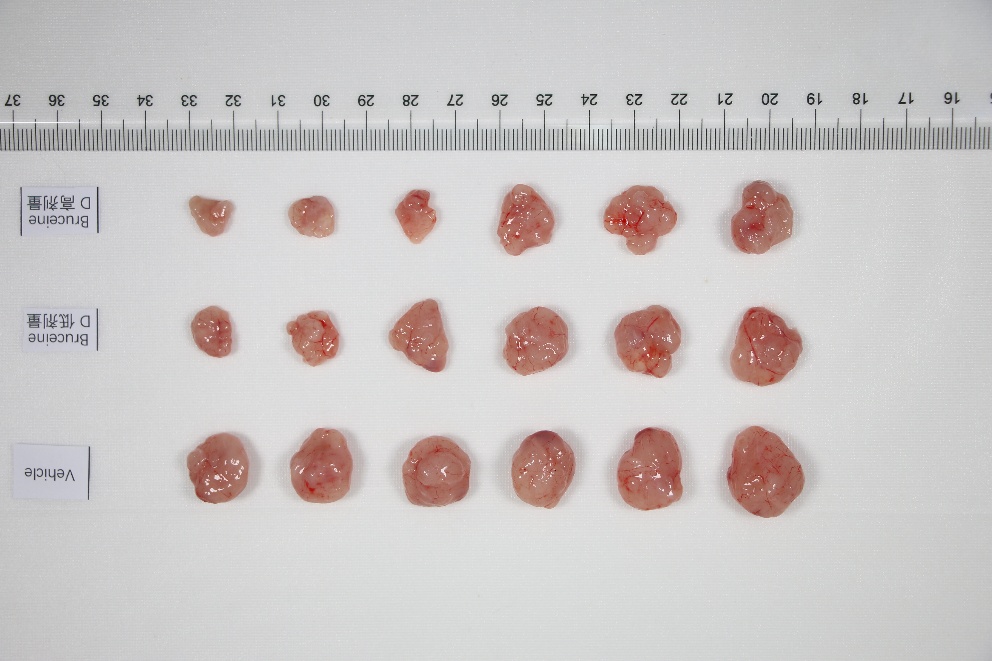

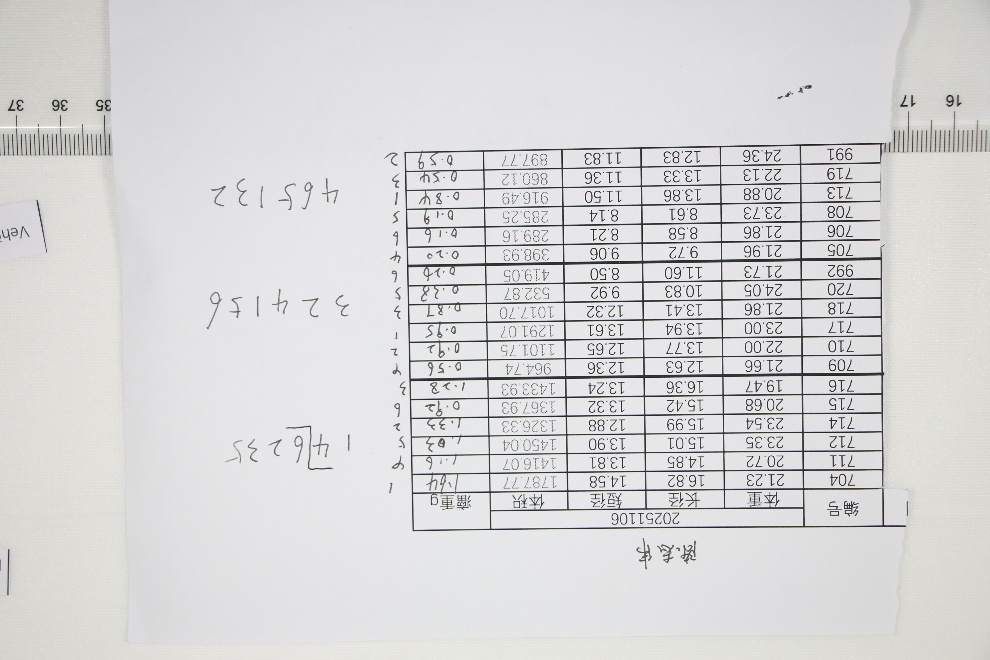


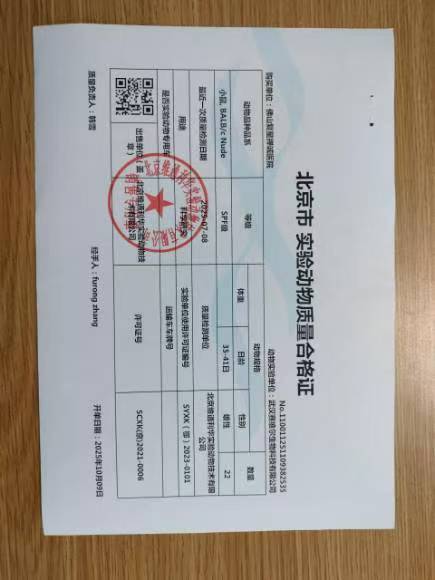


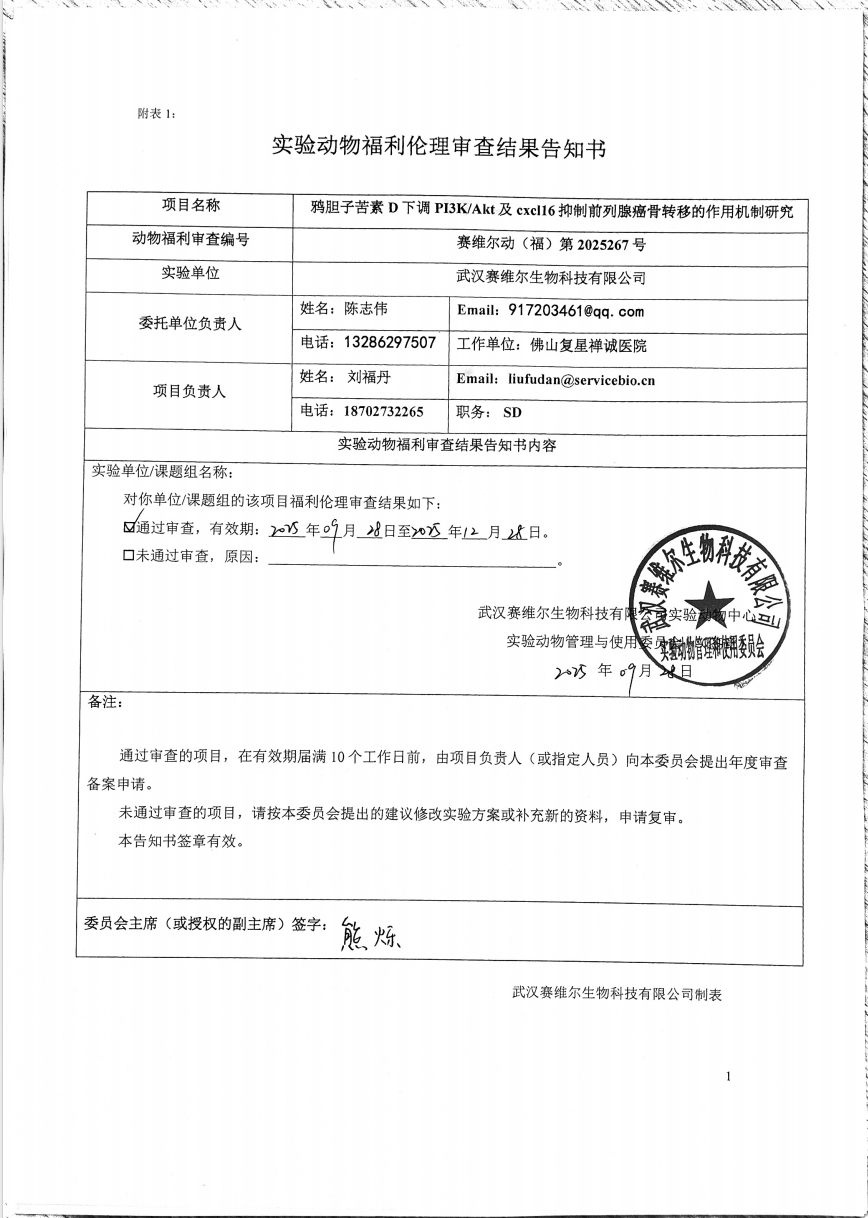


**Supplementary Figure2.RNA sequencing results(PCA analysis, correlation heatmap, and KEGG pathway enrichment results)**


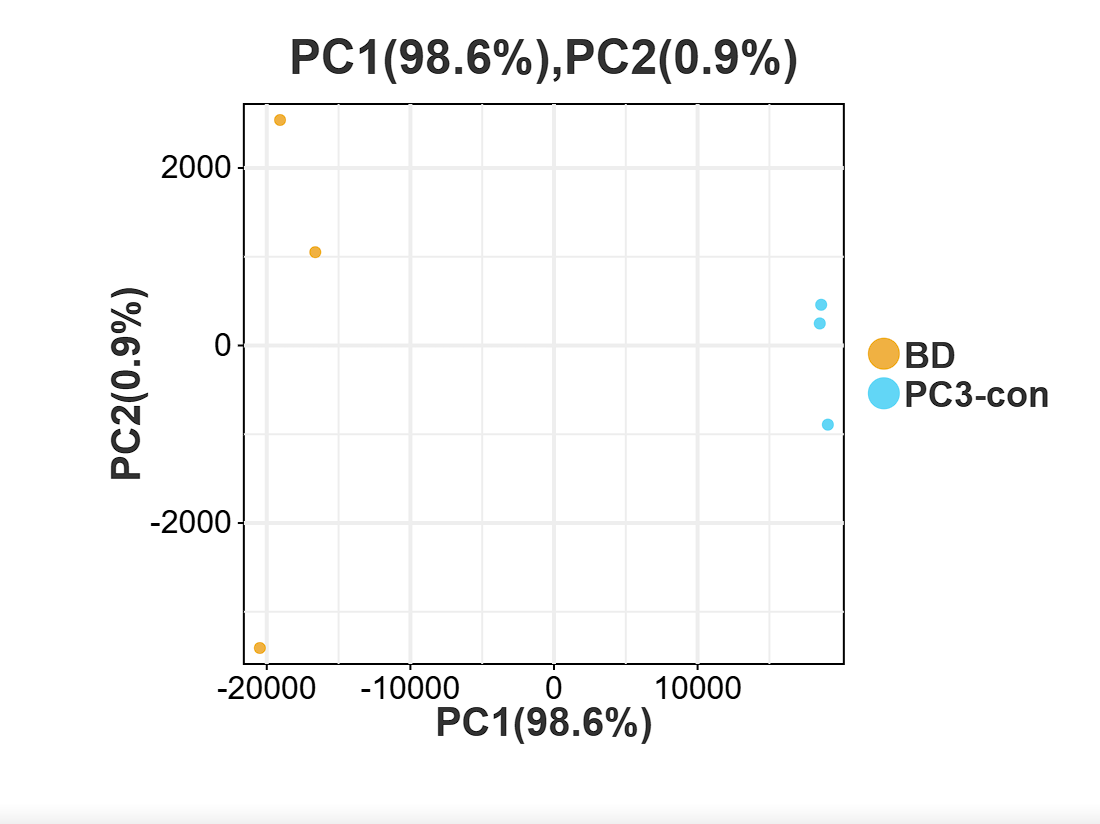


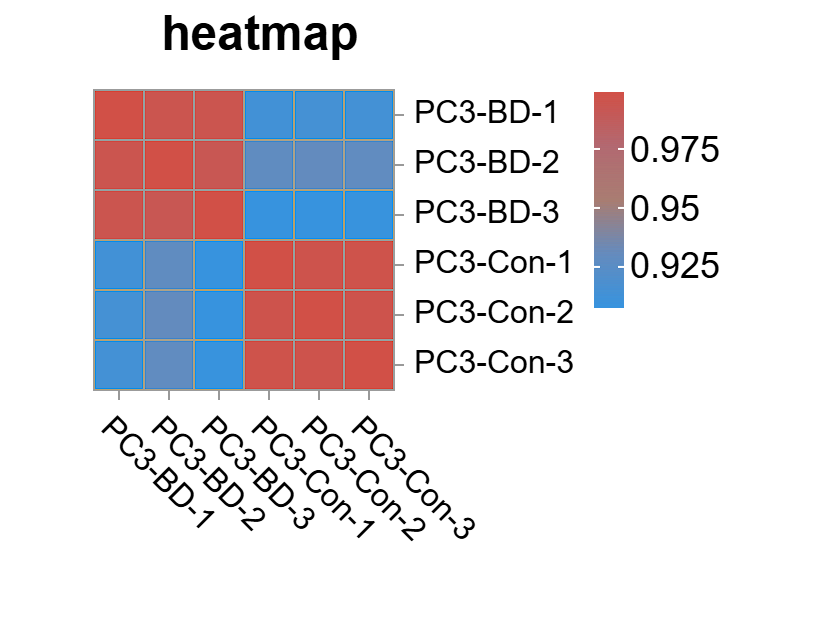

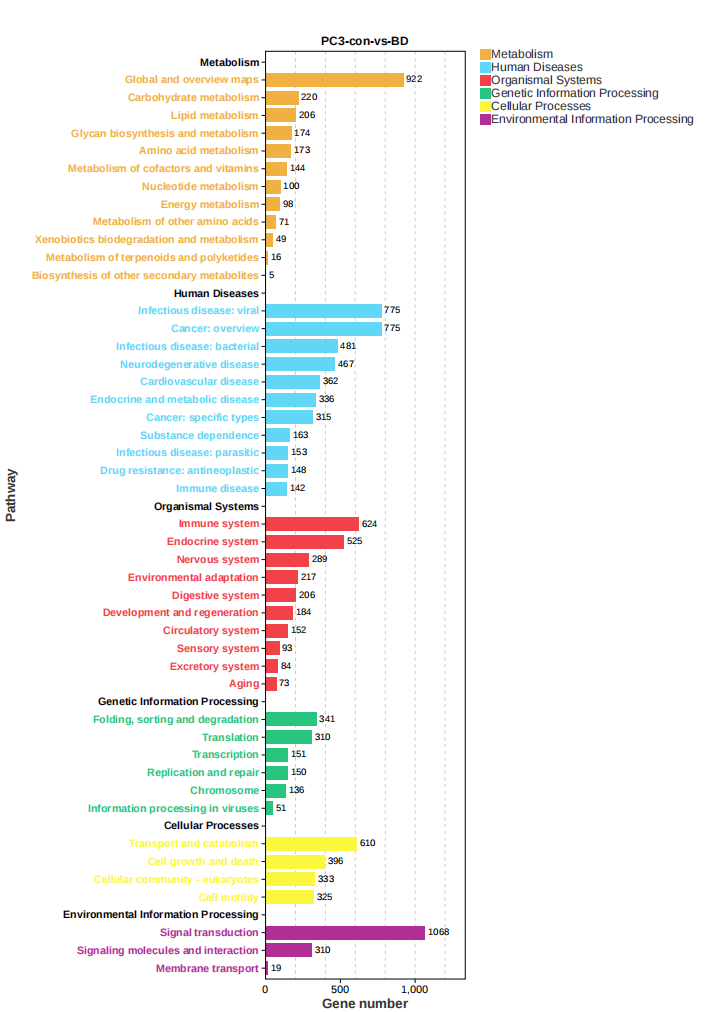

Supplement: Supplementary file 1 [file DataSheet1.zip › Supplementary Materials/Supplementary_Material.docx]
