# Supplementary material for: Bruceine D attenuates prostate cancer growth and motility by suppressing PI3K/AKT signaling and downregulating CXCL16: implications for skeletal metastasis in orthopedic oncology
Source: Front Pharmacol. 2026 Jun 1;17:1819854. doi: 10.3389/fphar.2026.1819854 (PMC13266102; doi:10.3389/fphar.2026.1819854)
Supplement: Supplementary file 1 [file DataSheet1.zip › Supplementary Materials/2.Medicine structure 3D.zip!/Molecular files and binding energy/cxcl16 Molecular files and binding energy/小分子绘图组图2Dppt CXCL16.pdf]

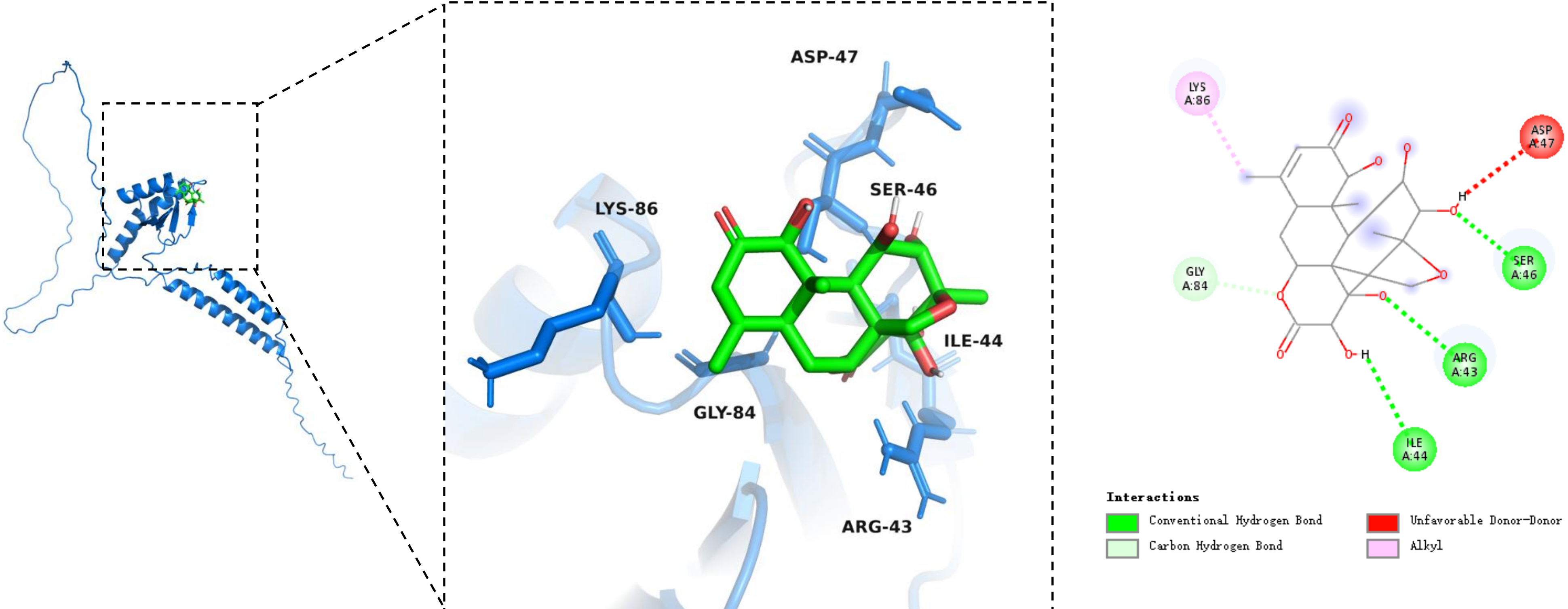
ASP-47
SER-46
LYS-86
ILE-44
GLY-84
ARG-43
LYS A:86
ASP A:47
GLY A:84
SER A:46
ARG A:43
ILE A:44
Interactions
Conventional Hydrogen Bond
Carbon Hydrogen Bond
Unfavorable Donor-Donor
Alkyl
