# Supplementary material for: Bruceine D attenuates prostate cancer growth and motility by suppressing PI3K/AKT signaling and downregulating CXCL16: implications for skeletal metastasis in orthopedic oncology
Source: Front Pharmacol. 2026 Jun 1;17:1819854. doi: 10.3389/fphar.2026.1819854 (PMC13266102; doi:10.3389/fphar.2026.1819854)
Supplement: Supplementary file 1 [file DataSheet1.zip › Supplementary Materials/2.Medicine structure 3D.zip!/Molecular files and binding energy.docx]

**TableS2 .** The binding energy of ligand Bruceine D to the receptor below。

| **Target** | Binding Energy（Kcal/mol） | Ki Dissociation constant μM |
| --- | --- | --- |
| AKT  PIK3AP1  PIK3CD  PIK3R2  CXCL16 | -6.891 | 8.826731642 |
|  | -6.83 | 9.784538326 |
|  | -9.129 | 0.201530174 |
|  | -6.431 | 19.19493582 |
|  | -7.025 | 7.03910913 |
|  |  |  |
