# Supplementary material for: Bruceine D attenuates prostate cancer growth and motility by suppressing PI3K/AKT signaling and downregulating CXCL16: implications for skeletal metastasis in orthopedic oncology
Source: Front Pharmacol. 2026 Jun 1;17:1819854. doi: 10.3389/fphar.2026.1819854 (PMC13266102; doi:10.3389/fphar.2026.1819854)
Supplement: Supplementary file 1 [file DataSheet1.zip › Supplementary Materials/3.PC-3Experimental data of PC-3 subcutaneous transplanted tumors 20251103.zip!/Pictures and license certificates related to the weight and tumor weight of mice in animal experiments.docx]

**Figure7.**
