# Supplementary material for: Bruceine D attenuates prostate cancer growth and motility by suppressing PI3K/AKT signaling and downregulating CXCL16: implications for skeletal metastasis in orthopedic oncology
Source: Front Pharmacol. 2026 Jun 1;17:1819854. doi: 10.3389/fphar.2026.1819854 (PMC13266102; doi:10.3389/fphar.2026.1819854)
Supplement: Supplementary file 1 [file DataSheet1.zip › Supplementary Materials/4.pc3 - n-RNA seq.zip!/pc3 - 新/GO富集/GO 富集分析 二级柱状图.pdf]

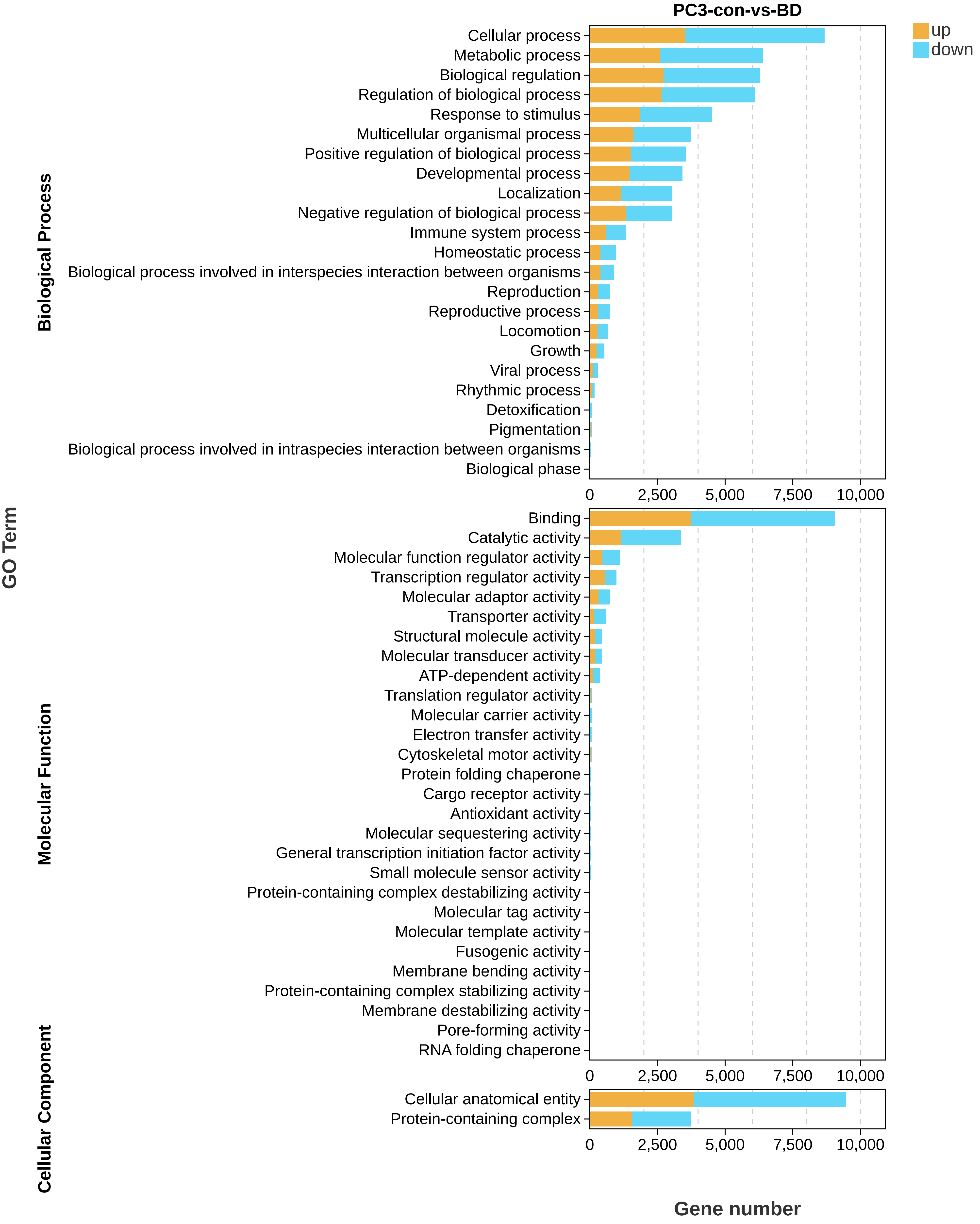
PC3-con-vs-BD
up
down
GO Term
Biological Process
Cellular process
Metabolic process
Biological regulation
Regulation of biological process
Response to stimulus
Multicellular organismal process
Positive regulation of biological process
Developmental process
Localization
Negative regulation of biological process
Immune system process
Homeostatic process
Biological process involved in interspecies interaction between organisms
Reproduction
Reproductive process
Locomotion
Growth
Viral process
Rhythmic process
Detoxification
Pigmentation
Biological process involved in intraspecies interaction between organisms
Biological phase
0
2,500
5,000
7,500
10,000
Molecular Function
Binding
Catalytic activity
Molecular function regulator activity
Transcription regulator activity
Molecular adaptor activity
Transporter activity
Structural molecule activity
Molecular transducer activity
ATP-dependent activity
Translation regulator activity
Molecular carrier activity
Electron transfer activity
Cytoskeletal motor activity
Protein folding chaperone
Cargo receptor activity
Antioxidant activity
Molecular sequestering activity
General transcription initiation factor activity
Small molecule sensor activity
Protein-containing complex destabilizing activity
Molecular tag activity
Molecular template activity
Fusogenic activity
Membrane bending activity
Protein-containing complex stabilizing activity
Membrane destabilizing activity
Pore-forming activity
RNA folding chaperone
0
2,500
5,000
7,500
10,000
Cellular Component
Cellular anatomical entity
Protein-containing complex
0
2,500
5,000
7,500
10,000
Gene number
