# Supplementary material for: Bruceine D attenuates prostate cancer growth and motility by suppressing PI3K/AKT signaling and downregulating CXCL16: implications for skeletal metastasis in orthopedic oncology
Source: Front Pharmacol. 2026 Jun 1;17:1819854. doi: 10.3389/fphar.2026.1819854 (PMC13266102; doi:10.3389/fphar.2026.1819854)
Supplement: Supplementary file 1 [file DataSheet1.zip › Supplementary Materials/4.pc3 - n-RNA seq.zip!/pc3 - 新/GO富集/RETU.pdf]

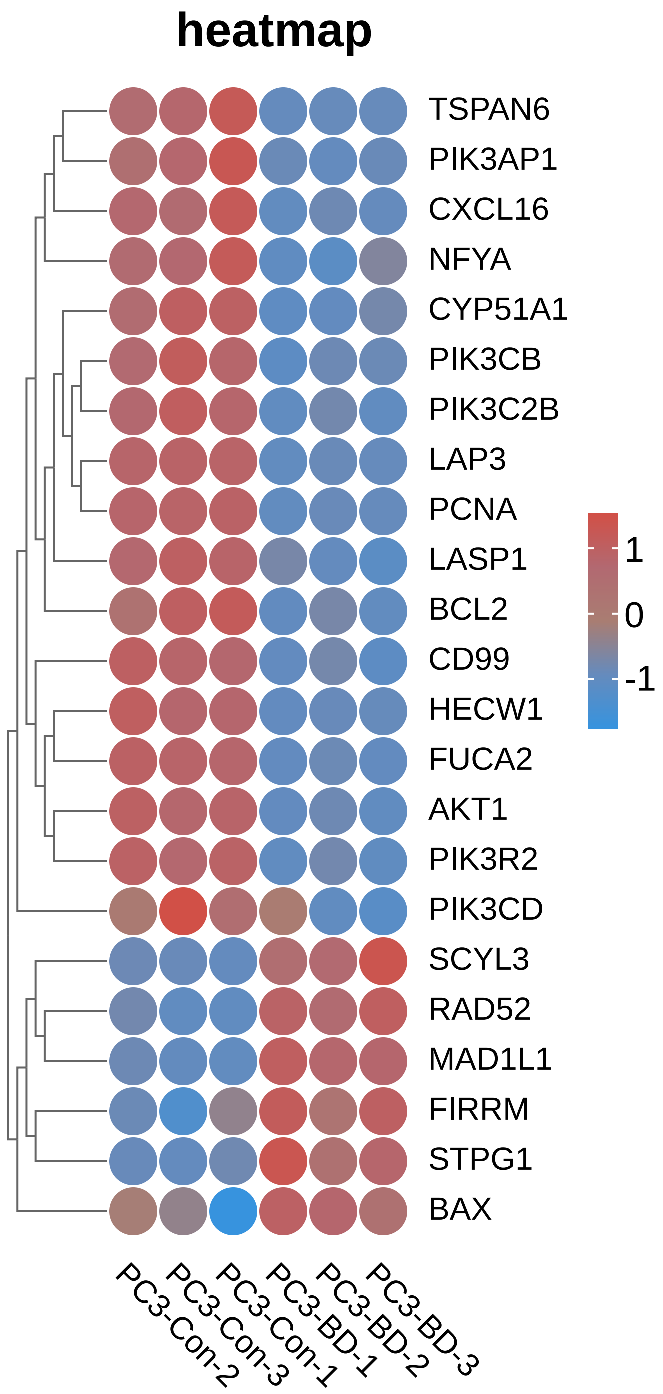
heatmap
TSPAN6
PIK3AP1
CXCL16
NFYA
CYP51A1
PIK3CB
PIK3C2B
LAP3
PCNA
LASP1
BCL2
CD99
HECW1
FUCA2
AKT1
PIK3R2
PIK3CD
SCYL3
RAD52
MAD1L1
FIRRM
STPG1
BAX
1
0
-1
PC3-Con-2
PC3-Con-3
PC3-Con-1
PC3-BD-1
PC3-BD-2
PC3-BD-3
