# Supplementary material for: Bruceine D attenuates prostate cancer growth and motility by suppressing PI3K/AKT signaling and downregulating CXCL16: implications for skeletal metastasis in orthopedic oncology
Source: Front Pharmacol. 2026 Jun 1;17:1819854. doi: 10.3389/fphar.2026.1819854 (PMC13266102; doi:10.3389/fphar.2026.1819854)
Supplement: Supplementary file 1 [file DataSheet1.zip › Supplementary Materials/4.pc3 - n-RNA seq.zip!/pc3 - 新/GSEA/PI3K-Akt signaling pathway.pdf]

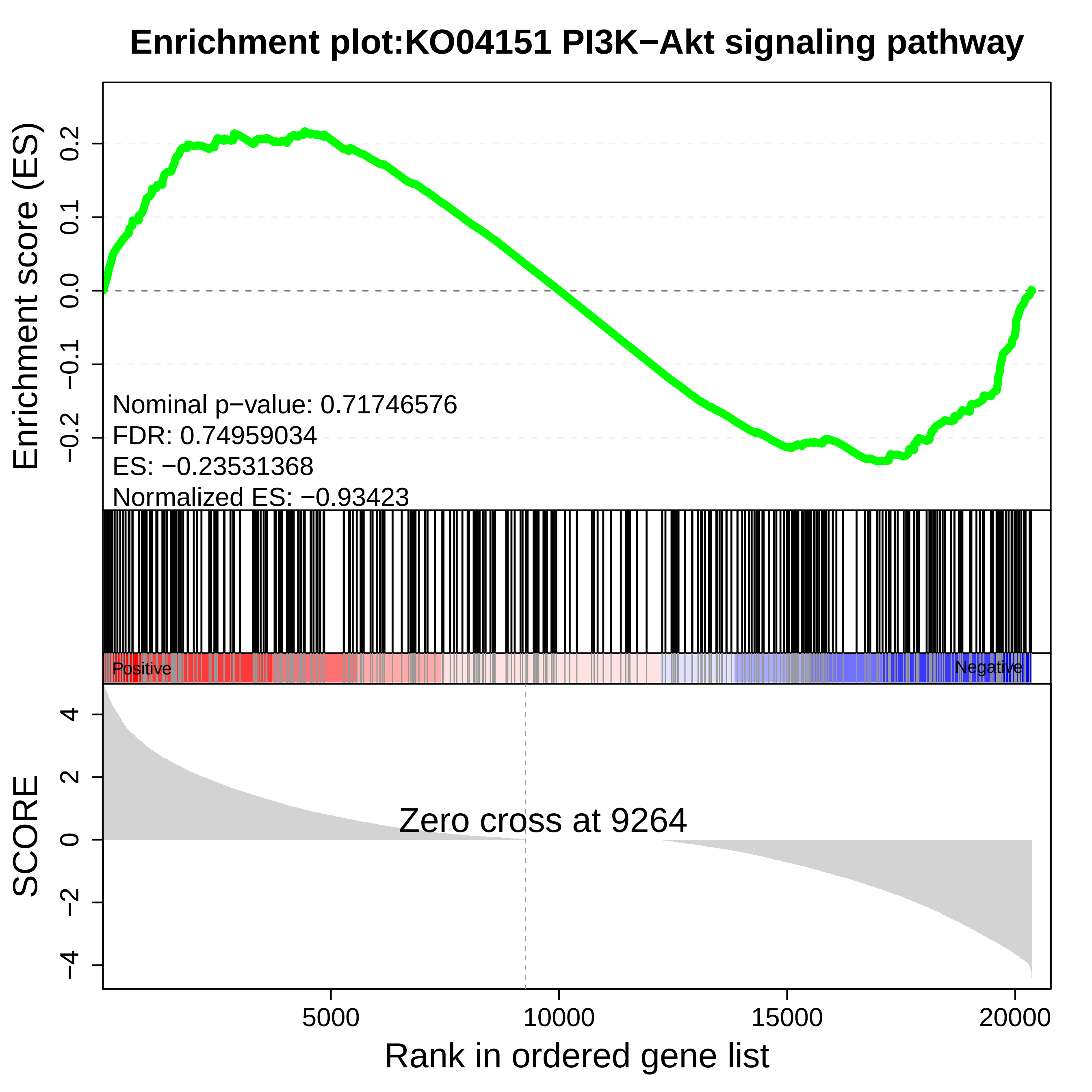
Enrichment plot:KO04151 PI3K−Akt signaling pathway
Enrichment score (ES)
0.2
0.1
0.0
−0.1
−0.2
Nominal p−value: 0.71746576
FDR: 0.74959034
ES: −0.23531368
Normalized ES: −0.93423
Positive
Negative
SCORE
4
2
0
−2
−4
Zero cross at 9264
5000
10000
15000
20000
Rank in ordered gene list
