# Supplementary material for: Bruceine D attenuates prostate cancer growth and motility by suppressing PI3K/AKT signaling and downregulating CXCL16: implications for skeletal metastasis in orthopedic oncology
Source: Front Pharmacol. 2026 Jun 1;17:1819854. doi: 10.3389/fphar.2026.1819854 (PMC13266102; doi:10.3389/fphar.2026.1819854)
Supplement: Supplementary file 1 [file DataSheet1.zip › Supplementary Materials/4.pc3 - n-RNA seq.zip!/pc3 - 新/KEGG富集/KEGG富集分析.pdf]

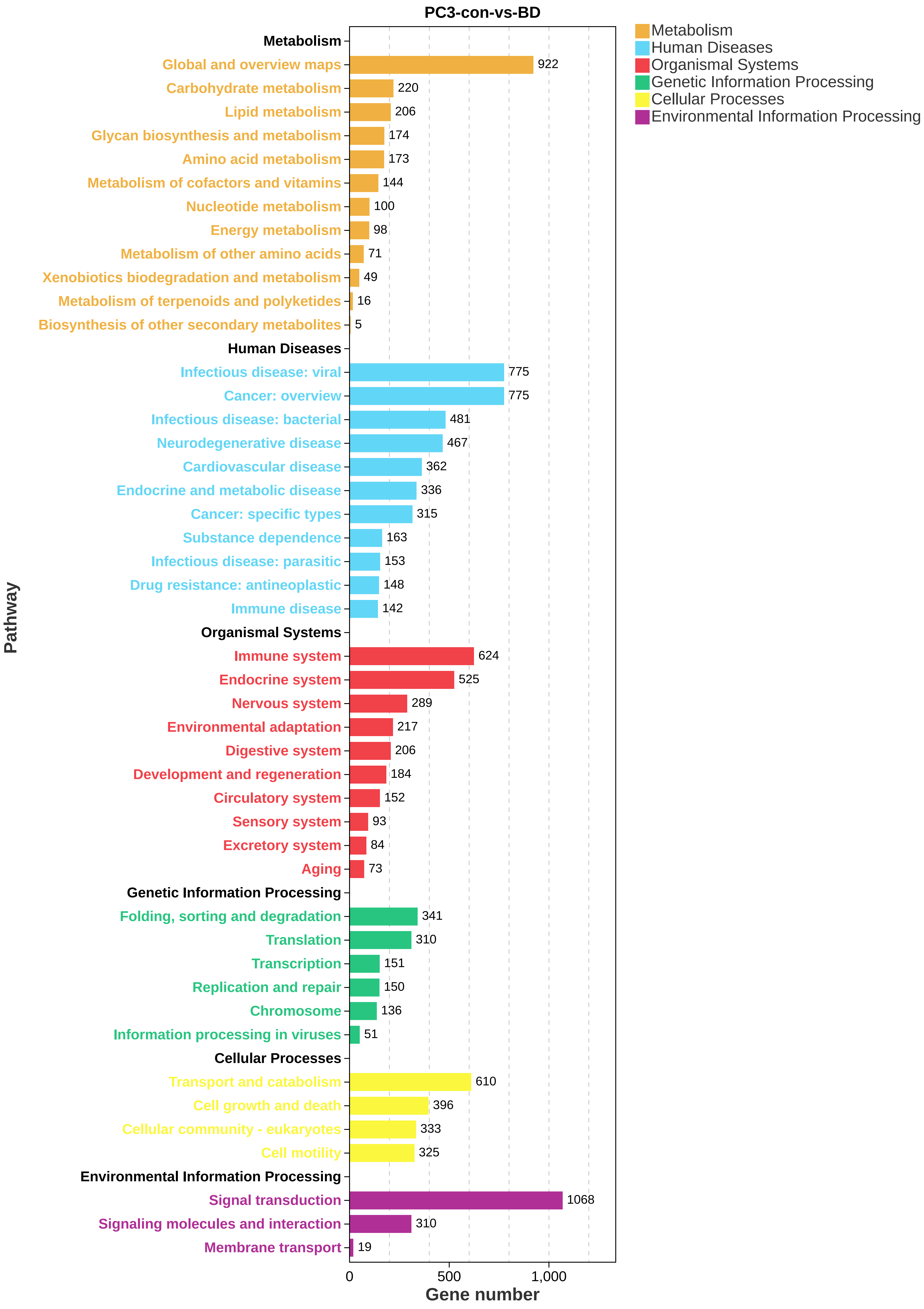

PC3-con-vs-BD
Metabolism
Human Diseases
Organismal Systems
Genetic Information Processing
Cellular Processes
Environmental Information Processing
Pathway
Metabolism
Global and overview maps
922
Carbohydrate metabolism
220
Lipid metabolism
206
Glycan biosynthesis and metabolism
174
Amino acid metabolism
173
Metabolism of cofactors and vitamins
144
Nucleotide metabolism
100
Energy metabolism
98
Metabolism of other amino acids
71
Xenobiotics biodegradation and metabolism
49
Metabolism of terpenoids and polyketides
16
Biosynthesis of other secondary metabolites
5
Human Diseases
Infectious disease: viral
775
Cancer: overview
775
Infectious disease: bacterial
481
Neurodegenerative disease
467
Cardiovascular disease
362
Endocrine and metabolic disease
336
Cancer: specific types
315
Substance dependence
163
Infectious disease: parasitic
153
Drug resistance: antineoplastic
148
Immune disease
142
Organismal Systems
Immune system
624
Endocrine system
525
Nervous system
289
Environmental adaptation
217
Digestive system
206
Development and regeneration
184
Circulatory system
152
Sensory system
93
Excretory system
84
Aging
73
Genetic Information Processing
Folding, sorting and degradation
341
Translation
310
Transcription
151
Replication and repair
150
Chromosome
136
Information processing in viruses
51
Cellular Processes
Transport and catabolism
610
Cell growth and death
396
Cellular community - eukaryotes
333
Cell motility
325
Environmental Information Processing
Signal transduction
1068
Signaling molecules and interaction
310
Membrane transport
19
0
500
1,000
Gene number
