# Supplementary material for: Bruceine D attenuates prostate cancer growth and motility by suppressing PI3K/AKT signaling and downregulating CXCL16: implications for skeletal metastasis in orthopedic oncology
Source: Front Pharmacol. 2026 Jun 1;17:1819854. doi: 10.3389/fphar.2026.1819854 (PMC13266102; doi:10.3389/fphar.2026.1819854)
Supplement: Supplementary file 1 [file DataSheet1.zip › Supplementary Materials/4.pc3 - n-RNA seq.zip!/pc3 - 新/差异基本分析/柱状图.pdf]

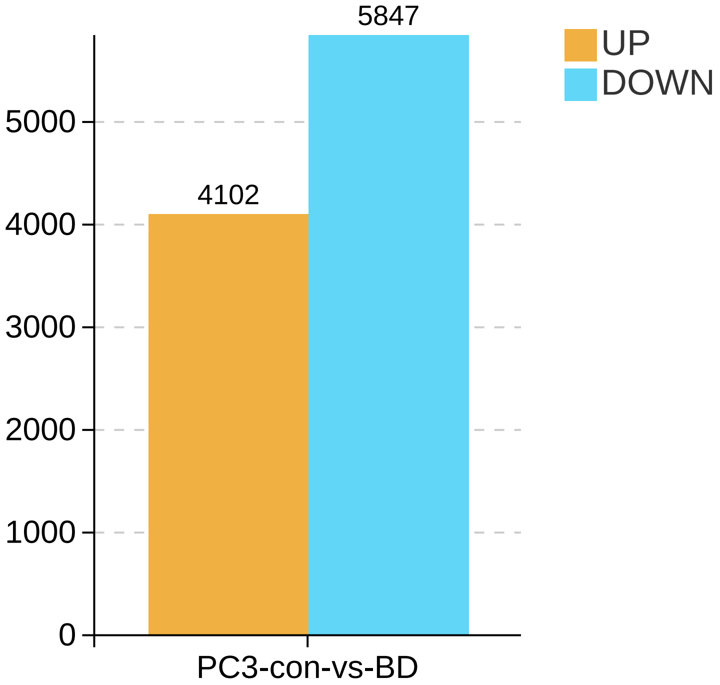
5847
4102
UP
DOWN
5000
4000
3000
2000
1000
0
PC3-con-vs-BD
